# Dental Hospitals

**Published:** 1910-03-15

**Authors:** Alice M. Steeves

**Affiliations:** Superintendent of the Hospital of Dentistry and Oral Hygiene, Boston, Mass.


### DENTAL HOSPITALS.

BY ALICE M. STEEVES, D.D.S.
Superintendent of the Hospital of Dentistry and Oral Hygiene, Boston, Mass.

Medical hospitals have been a blessing to the needy, a haven for the feeble-minded, and a source of much deprivation and discouragement to a large per cent. of the medical profession, because of the abuses and not of the legitimate and proper uses of institutions established for the alleviation of suffering. During the past fifty years private institutions have been established, and the hospital, for specialties, is found in every settlement boasting the dignity of town or city.

Dentistry, rejected by medicine in the beginning, has struggled for recognition by the mother profession, who in turn sneered, tolerated, and perhaps granted a corner or basement to the "tooth-carpenters," and until recently all this has been accepted by the majority of dentists as their portion.

However, a few progressive and undaunted souls have "kept the faith" and have worked steadily for the betterment of existing conditions. Now there is a great deal of writing and some talk clamoring for a place in the general hospital. All of this should have taken place twenty years ago. The time has now come when dental hospitals should be established either independently or as departments of already existing hospitals, and be accessible at all hours to those suffering from dental lesions.

It is in this very *fact* that our friend, the advertising man, gets his chief support. He is open early and late, smiling and cruel, while our strictly ethical friend works from nine until four, and leaves the "suffering devils" for the quack.

The dental hospital should be a *practical educator*, conducted in such a way as to educate the people to appreciate good dentistry and its *value.* Free hospital clinics only educate the people to look for the best that man can give for *nothing.*

Good dentistry and good medicine mean hard work, hard thinking and tired brain and body. Why should one class of people be expected to give all? Free treatment and much work is cheerfully given, but wholesale charity pauperizes the giver as well as the receiver. All these things should be considered in the management of the dental hospital, and each and every patient should be made to feel the *real value* of what they receive, if they pay for it or not; and all should be made to pay in proportion to their ability to pay.

If people want a piano, furs or other luxuries, they save and save, until they get it. But those priceless pearls, the teeth, that make or mar the human physiognomy, are never considered until aching pulps or inflamed gums drive them to the cheapest dentist. Consequently, heredity has furnished the material and *made* the orthodontist the necessity.

To sum up, the dental hospital should be an institution equipped for every department of dentistry, ready to receive patients at any time. The staff should consist of a superintendent and necessary assistants, together with a corps of interested consultants. Oral hygiene should be practiced "in season and out of season," and no patient should be allowed to leave without a clear understanding of what a dirty month means.—*Items of Interest.*

